# Risk factors for tooth loss in adults: A population-based prospective cohort study

**DOI:** 10.1371/journal.pone.0219240

**Published:** 2019-07-22

**Authors:** Manoelito Ferreira Silva Junior, Marília Jesus Batista, Maria da Luz Rosário de Sousa

**Affiliations:** 1 Department of Dentistry, State University of Ponta Grossa, Ponta Grossa, Paraná, Brazil; 2 Department of Community Health, Faculty of Medicine Jundiaí, Jundiaí, São Paulo, Brazil; 3 Department of Community Dentistry, Piracicaba Dental School, University of Campinas, Piracicaba, São Paulo, Brazil; Griffith University, AUSTRALIA

## Abstract

The aim of this study was to identify the risk factors for tooth loss in an extended age group of adults over 4 years. The prospective cohort study assessed adults (20–64 years old) in 2011 and 2015, from Piracicaba, São Paulo, Brazil. The sample selection was planned based on the adult population in the city. The inclusion criteria were randomly selected residences per census tract unit (one adult per household). The exclusion criteria comprised of a physical or psychological state that prevented the achievement of clinical procedures or understanding of the questionnaire. The home oral examination was performed using the index of decayed, missing, and filled teeth (DMFT), the Community Periodontal Index according to the World Health Organization, and visible biofilm. Demographic and socio-economic data, information on health habits, and the use of dental services were obtained by questionnaire. The outcome was a presence incidence of tooth loss, assessed by the difference between Missing teeth (M>0) from DMFT in 2011 and that in 2015. The conceptual theoretical model ‘Ethnicity, aging and oral health outcomes’ was adapted for tooth loss and used in a Hierarchical multivariate Poisson Regression analysis (p<0.20). The reference category for the Poisson regression were individuals who had no missing teeth (M) due to caries or periodontal disease (p<0.05). There were a total of 143 (follow-up rate = 57.7%) participants in the four-year study, and there was incidence of tooth loss in 51 (35.7%) adults over this period. The risk factors for tooth loss were reason for seeking dental services by pain (RR = 2.72; 95.0% CI: 1.04–7.37), previous tooth loss (RR = 3.01; 95.0% CI: 1.18–7.73) and decayed teeth (RR = 2.87; 95.0% CI: 1.22–6.73). The risk factors for tooth loss were: reason for seeking dental services by pain, previous tooth loss and dental caries.

## Introduction

In the past few decades, there has been a reduction in the prevalence and incidence of tooth loss in the world [1], but this condition is still amongst the top hundred conditions that had the highest impact on the health of the world population [2]. Tooth loss is an oral condition that leads to functional, aesthetic, and social damage, having an impact on the individual’s quality of life [3,4]. It is also associated with an increase in cardiovascular disease and risk of stroke [5].

Tooth loss is considered the most useful indicator of the general condition of oral health [6, 7], because it indicates the cohort effect of oral disease, both the individual’s and professional attitude and behaviour toward dental hygiene, the accessibility and philosophy of dental services [7–10], and also beliefs and cultural values about oral health. This fact demonstrates the relevance of studying tooth loss amongst the adult population, improving the understanding of the risk factors that lead to this condition besides social determinants, which are clearly proved to be a likely association to having more missing teeth.

The study deals with related factors associated with tooth loss, such as: age [7,11–13], sex [7,12,14,15], socio-economic status [11,14], residence in rural areas, lower educational level [7,12–14], more than five residents per household [13], use of public services [12,13], reason for seeking dental services [13], smoking, previous tooth loss, dental caries and periodontal disease [7,16,17]. Most population studies of adults use a cross-sectional design and measure associated factors [12,18–23], with few cohort studies [14,16,24] that have verified risk factors for tooth loss.

Cohort studies are still little explored in developing countries, as in Brazil, mainly due to the methodological difficulty to cover the same individuals [7]. The distribution of the diseases and their risk factors may be different according to the population studied. Therefore, cultural aspects of Brazil, whether of individual or professional origin, deserve better understanding. In addition, the study on dental losses in Brazil can assist in the evaluation of public health policies implemented in recent years, as well as in the planning of new public policies consistent with the health needs of the population studied [25].

No population cohort study on tooth loss in adults has been conducted in Brazil so far. The risk factors for tooth loss in this population are not yet fully understood. The objective of this study was to assess the risk factors for tooth loss in an extended age group of adults over four-year.

## Methods

### Design and ethical aspects

The present prospective four-year cohort study carried out in Piracicaba, São Paulo (SP), Brazil [26].

The present study was approved by the Ethics Research Committee of the Piracicaba Dental School, University of Campinas (Number 177/2009).

### Population and sample

Baseline. To calculate the representative sample of adults (20–64 years) living in Piracicaba, São Paulo, oral health conditions were assessed in different age groups and two different calculations were estimated for the sample size of young adults (20–44 years) and older adults (45–64 years). We adopted a design effect of 1.5, adopted a margin of error of 10.0%; and a 95.0% confidence interval, included data concerning the prevalence of cavities for each age group (70.2% and 90.9%, respectively [17]), and added 20.0% to the total to compensate for occasional losses. The sample size for adults aged 20–44 years was 172, and for those aged 45–64 years was 68, totalling 240 adults [26].

Sample selection was carried out in two stages and based on the Brazilian Demographic Census (2000)—the latest data compiled at the time in which the study was conducted. The adult population of Piracicaba between 20–64 years old was 202,131. In the first stage, the unit of selection was the census tract: from 456 census tracts, 30 were randomly selected (plus 2, in case substitutions were required). The second stage consisted of the selection of households, and a 30% increase in the probabilistic sample size to select the homes was used to compensate for non-responses. This resulted in a total of 342 houses, divided by the 30 census tracts selected for the study, resulting in a fraction of 11.4 houses per census tract. Based on the average population size of each census tract, 11 houses per tract were randomly selected and then one adult per house was also randomly selected [26].

The inclusion criteria was: being a Piracicaba resident aged 20–64 years old, with the mental capacity to answer the study questionnaire and agreeing to participate in the research. Mental capacity was evaluated by the subject’s capacity to comprehend the questions and answer properly during the interview and evaluation. The adult needed to be in appropriate physical condition to participate in the study [26]

Follow-up. The same subjects used in the baseline were considered for the follow-up study, without regard to where they were currently living (i.e., even those who had moved away were followed up) [26].

### Data collection

Baseline. Data collection took place between June and September 2011. The survey consisted of an oral examination and interview. The clinical oral examinations were performed in the subject’s home under natural light (without previous prophylaxis or drying) using Community Periodontal Index (CPI) probes and oral flat mirrors as recommended by the World Health Organization [27]. The clinical examination surveyed included coronal dental cavities measured by the decayed, missing and filled tooth index (DMFT), the periodontal condition of the CPI [27], and the presence of visible biofilm [28]. In addition, a questionnaire for the collection of demographic, socio-economic, health habit, and use of dental service information was provided at the baseline.

Only examiners trained by a benchmark examiner (trainer) were allowed to clinically survey subjects. Training included theoretical and practical discussions, which accounted for a total of 16 hours. Each examiner was trained to assess coronal cavities, periodontal condition, and visible biofilm. The intra-examiner agreement was observed ≥96.5% and the Kappa index ranged from 0.89 to 1.00 for all clinical conditions, which were both within the standards of reliability [29].

At the baseline, the sample was composed of 248 adults, representing the 149,635 adults (20–64 years old) living in Piracicaba, São Paulo, Brazil [26]. The refusal rate was 24.0%, because some individuals selected did not agree to participate in the study or could not be found during one of our three visits.

Follow-up. Data collection took place between June 2015 and September 2015. The same participants were sought at their addresses to participate in the study. Each participant signed a new Free and Clarified Consent Term to participate in the present study. The same clinical conditions were assessed, using the same criteria and examination protocol [26]. At the time of data collection, each individual retained the same baseline identification.

Two examiners participated in this stage of data collection; they were trained by a benchmark examiner (baseline examiner) with theoretical and practical discussions, calibrated to a total of 20 hours. The examiners were trained to assess coronal cavities, periodontal condition and visible biofilm. The intra-examiner agreement was ≥ 96.5% and inter-examiner agreement was ≥ 90.0%, and the Kappa index ranged from 0.89 to 1.00 for all clinical conditions, which was within the standards of reliability [29].

### Study variables

The dependent variable was incidence of tooth loss was stratified into codes 0 (no missing) and 1 (missing ≥ 1 teeth in 4 years) assessed by the difference between the Missing teeth (M) and the Decayed, Missing, and Filled Teeth index (DMFT) in 2011 and 2015. Missing teeth (M) were considered the teeth with codes 4 (tooth loss due to dental caries experience) and 5 (tooth loss due to other causes) of the DMFT index. To calculate the clinical variables, 32 teeth were considered.

**Fig 1:**
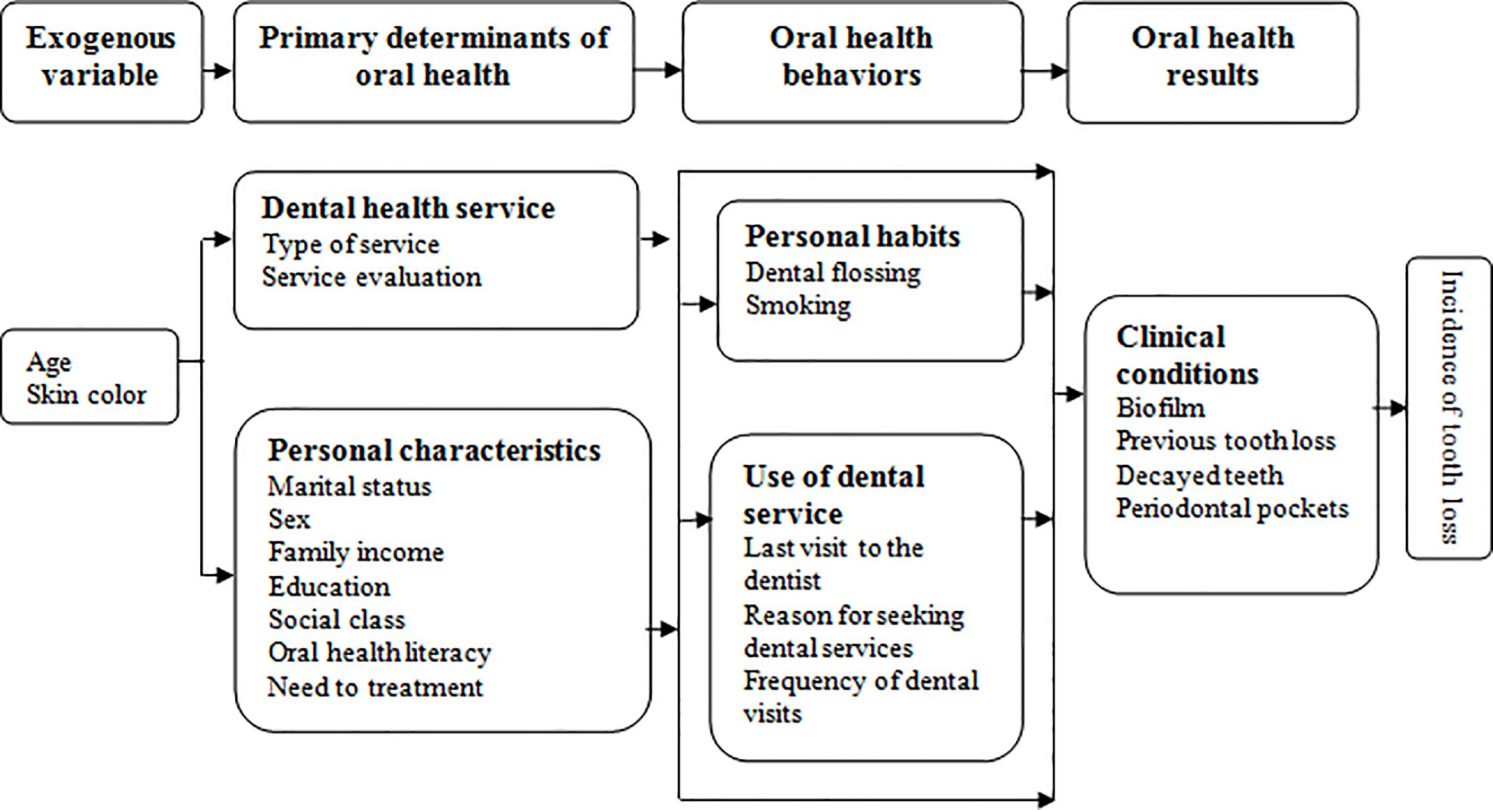
Theoretical conceptual model of tooth loss adapted for the study (Andersen and Davidson, 1997). Piracicaba (SP), Brazil, 2015.

The independent variables measured were related to baseline data and divided into four blocks (Fig 1). In the first block, an exogenous variable age was separated into categories of young adults (20–44 years), older adults (45–64 years) and skin color groups were defined by self-declaration, and these were categorized as white and non-white (black, brown, yellow, or indigenous). In the second block, primary determinants of oral health assessed the type of dental service (public, private, or insurance), service evaluation (good or regular/bad), marital status (stable relationship or no stable relationship), sex (male or female), family income as multiples of the minimum wage (MW): high (≥ 3 MW), medium (1–2 MW), or low (≤1 MW), and education level (≤4 years, between 5–10 years, and ≥11 years). Social class was assessed according to the classification of Graciano et al. [30], which uses a score based on education, family income, occupation, type of residence, and number of residents at a household, gathering the scores into six social classes. For the present study, classes were divided into three groups: high, medium and low. Regarding information on oral health, oral health literacy was assessed using the method of Ishikawa et al. [31] for high or low, while the need for treatment was classified as ‘yes’ or ‘no’.

In the third block, behaviours in oral health, personal health practices, and dental service utilization were assessed, such as: dental flossing (usual or unusual), smoking (yes or no), time since last visit to the dentist (<1 year, 1–2 years, and >2 years), reason for seeking dental services (routine, need, and pain), and frequency of dental visits (regularly or non-regular). In the fourth block, oral health outcomes were assessed, with the analysis being: presence of visible dental biofilm considered ‘yes’ (for at least one tooth surface with visible dental biofilm) or ‘no’ (all tooth surfaces without visible dental biofilm); Previous tooth loss was created with baseline data of missing teeth dichotomized in the median that was five teeth (more than 5 tooth loss and 4 or less tooth loss); Decayed teeth it was considered ‘yes’ (or present) if the individual had at least one decayed tooth, or ‘no’ (absence of decayed teeth); and size of the periodontal pocket, being considered ‘yes’ for those with at least one sextant presenting clinical attachment loss (CAL) more than 4 mm (Code 3 and 4 of the index).

### Data analysis

**Fig 2:**
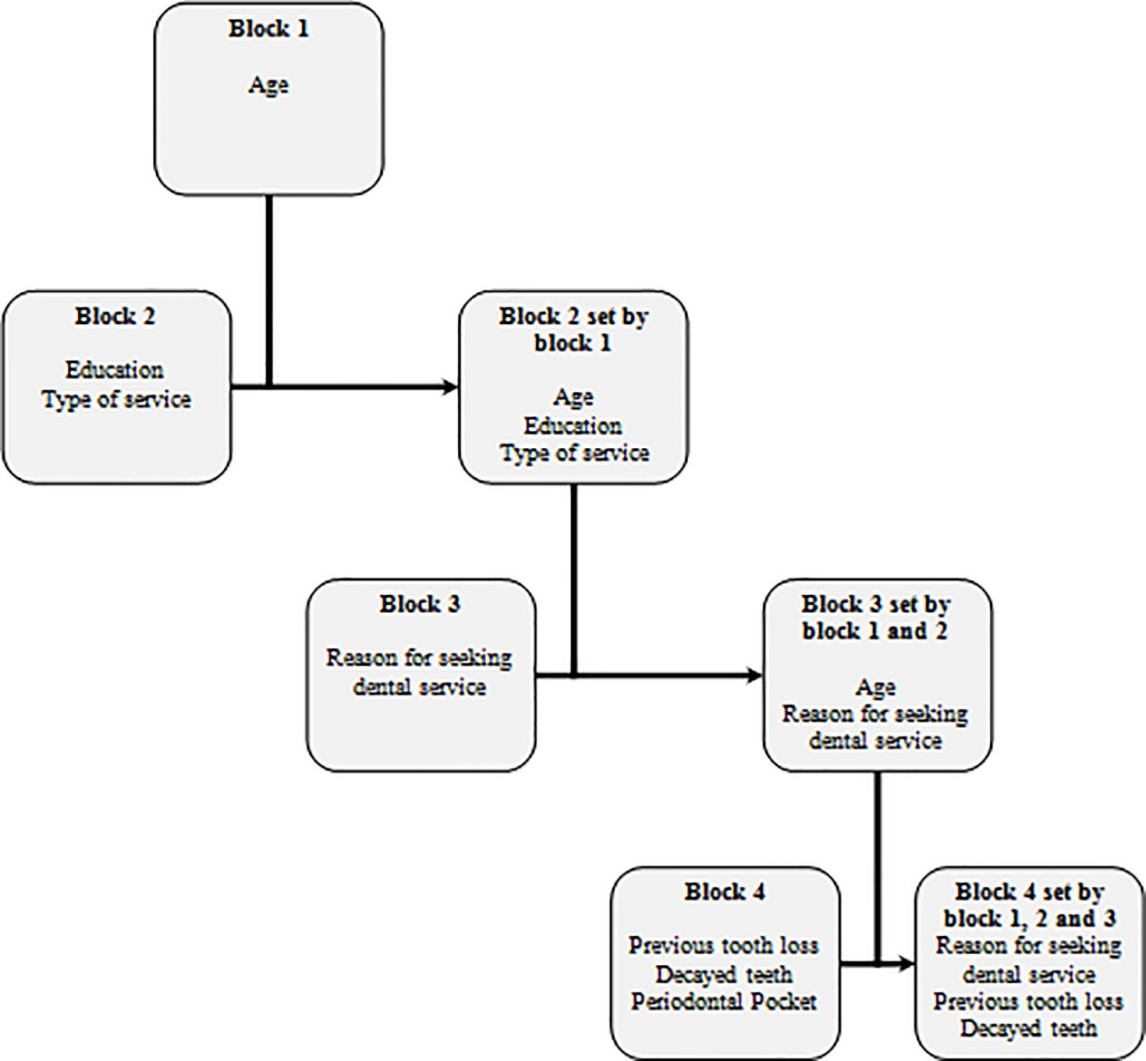
Schematic illustration of the modelling process of hierarchical Poisson regression. Piracicaba (SP), Brazil, 2015.

Data were tabulated using IBM SPSS Statistical version 20.0 and Excel. A conceptual theoretical model adapted from “Ethnicity, aging and oral health outcomes” proposed by Andersen and Davidson [32] was adapted for the tooth loss study (Fig 1). This model was used to direct the hierarchical analysis [33] into four blocks considering exogenous variables, primary determinants of oral health, oral health behaviour and oral health results. Univariate analysis of Poisson regression was performed between the outcome that was the incidence of tooth loss and independent variables of the theoretical conceptual model. Multivariate Poisson Regression was performed in order the prevalence of tooth lost higher than 20% in this population. Each block was adjusted separately and the variables with a value of p < 0.20 were selected to adjust the model. The variables that remain significant in each block, as described in Fig 2, adjusted the model following the hierarchical approach: Block 1 adjusted block 2, and the result obtained adjusted the subsequent block, until the final model was obtained. The reference category for the Poisson Regression was adults had no missing teeth (M) due to caries or periodontal disease (p < 0.05). The β coefficient was interpreted as Relative Risk (RR). Hosmer-Lemeshow goodness of fit was performed.

## Results

After a four-year follow-up, a total of 143 adults (follow-up rate = 57.7%) constituted the sample. The sample was composed mainly of women showing the following characteristics: older adults in a stable relationship with a higher family income and education level equal or up to 11 years (Table 1).

**Table 1:** Bivariate analysis of demographic and socioeconomic characteristics between adult participants and non-participants in Piracicaba, Brazil, 2015.

			2011	2015		
Characteristics			Total sample	Non-participants	Participants	
			n (%)	n (%)	n (%)	p-value*
Demographics**	*Sex*	Male	69 (27.8)	29 (27.6)	40 (28.0)	0.534
		Female	179 (72.2)	76 (72.4)	103 (72.0)	
	*Age*	Young adults	138 (55.6)	68 (64.8)	70 (49.0)	0.013
		Older adults	110 (44.4)	37 (35.2)	73 (51.0)	
	*Skin color*	White	198 (79.8)	76 (72.4)	122 (85.3)	0.012
		No white	50 (20.2)	29 (27.6)	21 (14.7)	
	*Marital status*	Stable relationship	174 (70.2)	62 (59.0)	112 (78.3)	0.001
		No stable relationship	74 (29.8)	43 (40.1)	31 (21.7)	
Socio-economics**	*Family Income*	Low	39 (15.7)	16 (15.2)	23 (16.1)	
		Medium	161 (64.9)	70 (66.7)	91 (63.6)	0.920
		High	42 (17.0)	17 (16.2)	25 (17.5)	
		Missing data	06 (2.4)	02 (1.9)	04 (2.8)	
	*Education*	≤ 4 years	43 (17.4)	13 (12.4)	30 (21.0)	0.200
		5–10 years	69 (27.8)	32 (30.5)	37 (25.9)	
		≥ 11 years	136 (54.8)	60 (57.1)	76 (53.1)	
	*Social class*	Low	38 (15.3)	15 (14.3)	23 (16.1)	0.909
		Medium	167 (67.4)	71 (67.6)	96 (67.1)	
		High	43 (17.3)	19 (18.1)	24 (16.8)	

*Chi-square test (p<0.05)

**Baseline (2011) data were used as reference.

After four years, incidence of tooth loss occurred in 51 (35.7%) adults, totalling 130 missing teeth. The mean cumulate incidence of tooth loss was 0.91 (SD = 1.65) per adult.

Fig 2 shows the hierarchical modelling process performed in the present study. Age was significant when adjusted in block 1. Education and type of service were significant when block 2 was adjusted. These three variables set block 3, resulting in the selection of age, type of service and reason for seeking dental services, which set block 4, producing the final model (Table 2). After the hierarchical analysis, reason for seeking dental services by pain (RR = 2.72; 95.0% CI: 1.04–7.37), previous tooth loss (RR = 3.01; 95.0% CI: 1.18–7.73) and decayed teeth (RR = 2.87; 95.0% CI: 1.22–6.73) showed an adjusted relative risk for tooth loss.

**Table 2:** Univariate and Hierarchical Multivariate Poisson Regression analysis, crude and adjusted relative risk (RR) and 95.0% confidence intervals (CI) of incidence of tooth loss and demographic, socio-economic and health practice characteristics of adults living in Piracicaba, Brazil, 2011–2015.

	No incidence	Incidence ≥ 1 teeth	Total	Crude RR		Adjusted RR					
						Model 1		Model 2		Model 3	
Variables	n (%)	n (%)	n (%)	RR (95.0%CI)	p- value	RR (95.0%CI)	p-value	RR (95.0%CI)	p-value	RR (95.0%CI)	p-value
***Exogenous variable***											
**Age**											
Young adults	53 (75.7)	17 (24.3)	70 (100.0)	1		1		1		1	
Older adults	39 (53.4)	34 (46.6)	73 (100.0)	1.92 (1.18–3.10)	0.008	1.78(1.09–2.93)	0.022	1.64(1.03–2.61)	0.038	1.58 (0.61–4.13)	0.348
**Skin color**											
White	79 (64.8)	43 (35.2)	122 (100.0)	1							
Non-white	13 (61.9)	8 (38.1)	21 (100.0)	1.13 (0.44–2.94)	0.801						
***Oral health determinants***											
Type of dental service*											
Private	40 (59.7)	27 (40.3)	67 (100.0)	1		1		1			
Insurance	32 (80.0)	08 (20.0)	40 (100.0)	0.47 (0.25–0.98)	0.045	0.53 (0.28–1.04)	0.064	0.53(0.27–1.04)	0.066		
Public	18 (52.9)	16 (47.1)	34 (100.0)	1.17 (0.74–1.85)	0.509	1.37 (0.86–2.16)	0.183	1.18(0.77–1.80)	0.456		
**Service evaluation**											
Good	78 (64.5)	43 (35.5)	121(100.0)	1							
Regular/Bad	12 (60.0)	08 (40.0)	20 (100.0)	1.17 (0.62–2.03)	0.693						
**Marital status**											
No stable relationship	23 (74.2)	8 (25.8)	31 (100.0)	1							
Stable relationship	69 (61.6)	43 (38.4)	112(100.0)	1.49 (0.78–2.82)	0.225						
**Sex**											
Female	70 (68.0)	33 (32.0)	103 (100.0)	1							
Male	22 (55.0)	18 (45.0)	40 (100.0)	1.40 (0.90–2.19)	0.133						
**Family income**											
High	75 (64.7)	41 (35.3)	116 (100.0)	1							
Medium	11 (68.8)	5 (31.2)	16 (100.0)	0.88 (0.41–1.90)	0.753						
Low	3 (42.9)	4 (57.1)	7 (100.0)	1.62 (0.81–3.21)	0.171						
**Education**											
≥ 11 years	55 (72.4)	21 (27.6)	76 (100.0)	1		1		1			
5–10 years	22 (59.5)	15 (40.5)	37 (100.0)	1.47 (0.86–2.50)	0.159	1.34 (0.80–2.24)	0.274	1.16 (0.71–1.90)	0.554		
≤ 4 years	15 (50.0)	15 (50.0)	30 (100.0)	1.81 (1.09–3.01)	0.023	1.42 (0.84–2.42)	0.191	1.23 (0.74–2.05)	0.416		
**Social class**											
High	20 (83.3)	4 (16.7)	24 (100,0)	1							
Medium	59 (61.5)	37 (38.5)	96 (100.0)	2.31 (0.91–5.86)	0.077						
Low	13 (56.5)	10 (43.5)	23 (100.0)	2.61 (0.95–7.15)	0.062						
**Oral health literacy**											
High	46 (67.6)	22 (32.4)	68 (100.0)	1							
Low	46 (61.3)	29 (38.7)	75 (100.0)	0.84 (0.54–1.31)	0.434						
**Need of dental treatment**											
No	32 (76.2)	10 (23.8)	42 (100.0)	1							
Yes	60 (59.4)	41 (40.6)	101 (100.0)	1.70 (0.94–3.08)	0.076						
***Personal habits***											
**Dental flossing**											
Usual	33 (67.4)	16 (32.6)	49 (100.0)	1							
Unusual	59 (62.8)	35 (37.2)	94 (100.0)	1.14 (0.70–1.84)	0.592						
**Smoking**											
No	78 (65.0)	42 (35.0)	120 (100.0)	1							
Yes	14 (60.9)	9 (39.1)	23 (100.0)	1.12 (0.64–1.97)	0.699						
***Use of dental service***											
**Last appointment**											
< 1 year	58 (67.4)	28 (32.6)	86 (100.0)	1							
1–2 years	15 (55.6)	12 (44.4)	27 (100.0)	1.36 (0.81–2.30)	0.241						
≥ 3 years	19 (63.3)	11 (36.7)	30 (100.0)	1.13 (0.64–1.97)	0.678						
Reason for seeking dental services*											
Routine	55 (77.5)	16 (22.5)	71 (100.0)	1				1		1	** **
Need	17 (50.0)	17 (50.0)	34 (100.0)	2.22 (1.28–3.83)	0.004			1.90 (1.10–3.29)	0.021	2.20 (0.85–5.69)	0.104
Pain	15 (45.5)	18 (54.5)	33 (100.0)	2.42 (1.42–4.12)	0.001			2.04 (1.22–3.42)	0.077	**2.72 (1.04–7.37)**	**0.048**
Frequency of dental visits*											
Regularly	51 (73.9)	18 (26.1)	69 (100.0)	1							
Non-regularly	40 (54.8)	33 (45.2)	73 (100.0)	1.73 (1.08–2.77)	0.022						
***Clinical conditions***											
Dental biofilm*											
No	56 (66.7)	28 (33.3)	84 (100.0)	1							
Yes	31 (57.4)	23 (42.6)	54 (100.0)	1.28 (0.83–1.97)	0.267						
**Previous tooth loss (median)**											
4 ≤ teeth loss	60 (78.9)	16 (21.1)	76 (100.0)	1						**1**	
5≥ teeth loss	32 (47.8)	35 (52.8)	67 (100.0)	4.10 (1.98–8.52)	<0.001					**3.01 (1.18–7.73)**	**0.022**
**Decayed teeth**											
No	71 (74.0)	25 (26.0)	96 (100.0)	1						**1**	** **
Yes	21 (44.7)	26 (55.3)	47 (100.0)	2.12 (1.39–3.25)	<0.001					**2.87 (1.22–6.73)**	**0.015**
**Periodontal pocket**											
< 4 mm	73 (73.0)	27 (27.0)	100 (100.0)	1							
≥ 4 mm	19 (44.2)	24 (55.8)	43 (100.0)	2.07 (1.36–3.14)	0.001						

Goodness of fit of final model: Likelihood Ratio Chi-Square = 31,341 (p<0,001).

*Variables do not represent a total of 143 individuals as lost data were excluded from statistical analysis.

Model 1: Block 2 set by block 1. Model 2: Block 3 set by blocks 1 and 2. Model 3: Block 4 set by blocks 1, 2 and 3.

Note: The reference category for the Poisson regression analysis was individuals who showed no incidence of tooth loss in the past four years.

## Discussion

In our study, age, reason for seeking dental services by pain, previous tooth loss and decayed teeth were risk factors for tooth loss in adults. These risk factors reinforce that tooth loss is a result of progression and accumulation of the need for cavity treatments and could have been avoided. A population-based cohort study of tooth loss in adults provides more detailed information when compared to the cross-sectional studies on risk factors for incidence of tooth loss, and can contribute to planning and actions to promote oral health.

The present study has some limitations. As with other cohort studies [14,16,24], there was a loss of follow-up in the present study, however, the profile was maintained of the initial sample, verified by the distribution by sex, income, education level and social class, despite the loss of the sample. It is expected that maintenance participation of individuals with stable demographic characteristics will be maintained in the household study, particularly those with their own homes, stable relationships, are older, and have white skin, who typically benefit from the best socio-economic conditions among adults due to the large racial inequality in Brazil, a reality also found in other Brazilian cities. In this longitudinal study, a new sample calculation was not performed, because the objective was to be able to evaluate dental loss in the same individuals from the initial sample. Regarding the presence of periodontal pocket, it should be noted that this measure was performed by index tooth in each sextant, and not individualized, which may hamper the association of the periodontal condition with the incidence of tooth loss.

There has been a reduction in the prevalence of tooth loss in adults worldwide, although the incidence has not been reduced for all age groups. In particular, adults between 30 and 50 years of age did not show a decrease in tooth loss between 1990 and 2010 [1]. The prevalence of tooth loss shows a tendency to increase according to the age group of the individuals studied, and the incidence initiated mainly in adults remains stable over the life of the individual [34]. An association between age and tooth loss is established in scientific literature [1,11–13,16,24], although the process of physiological ageing does not justify the degree of tooth loss [35]. Therefore association between age and tooth loss is established in scientific literature [1,7,9,11–13,16,24], but the process of physiological ageing does not justify the degree of tooth loss [35].

Although of tooth loss prevalence increases gradually with age, showing a steep increase around 70 years of age, associated with a peak in incidence at 64 years of age [1], when the severity and accumulation of tooth loss throughout life reduces the total number of teeth in the oral cavity, and the increases the chance of the loss of remaining teeth. Therefore, studies have pointed out that there is a strong association of previous dental loss as a predictor of new dental losses, and it is not only due to the existence of a previous oral health condition, but it may indicate that the individual or dentist prefers tooth extraction as the choice treatment in the future. [7]. This understanding is reinforced in the present study, because adults that presented loss above the median in the baseline data had more risk to lost their teeth during the cohort follow-up. Previous study showed that the main reason for the extraction was dental pain aggravated by the understanding of the lack of another treatment option for the tooth [36]. Actually, present a previous tooth loss can demonstrate aspects that go beyond of clinical characteristics. In fact, is very important improve the comprehension of the tooth loss, because studies have shown associated factors and risk indicators, but measure behavior and beliefs is not easy work.

In the present study, tooth loss was determined by the late demand for dental care, that is, motivated by pain, as reported in Brazilian cross-sectional studies in adults [13,22]. This result can be explained by Brazilian oral health, particularly in the adult group, marked by experiencing difficulty in accessing dental health care and also a high demand for dental health care [36]. In addition, the overdue search for the treatment of oral diseases and their progression resulted in the need for invasive procedures and mutilating techniques in the recent past [8,10]. As a consequence, the dental office and dental surgeon remain unattractive options of treatment amongst most people. An additional important consideration is how to facilitate a routine search for dental services in an economically active population. The opening hours of dental health units appear to be a strong disincentive, as the majority only operate during business hours, which affect workers due to their inability to seek dental care during working hours [37].

However, it should be noted that different generations included in the adult age group reflect a history of professional practice and also of Brazilian public oral health policies. In Brazil, adults aged 45–64 years received no public oral health care, adults aged 35–44 years lived during a period in which the Brazilian National Institute of Medical Assistance and Welfare was created and reflect the beginning of the state’s concern with health, but with little oral health activity and dental practice remaining based on the model of restorative surgery [25]. During the 1980s fluoridation was implemented in Brazilian cities, thus, most participants between the ages of 20 to 34 benefited from the presence of fluoride in the drinking water. In addition, the National Health Service was consolidated in 1988 and oral health recognized as an integral part of general health and a human right, with a further expansion from the 2000s onwards [25]. Understanding how the Brazilian adult population has absorbed public health policies over time can help us in understanding the risk factors for tooth loss.

A few published studies exist on multilevel analysis that demonstrate an association between tooth loss and clinical conditions of oral health [19,22,23]. Our population-based cohort study is the first to verify dental cavities as a risk factor for tooth loss and also previous tooth loss. This result shows that the condition of tooth loss follows the progression and accumulation of requirements for cavity treatment [7,38,39]. However, despite advances in public health worldwide, untreated caries in permanent teeth remain the most prevalent disease among the world population [39]. Thus, it is impossible to think of reducing tooth loss without investing in effective public policies dealing with prevention and treatment in the early stages of dental cavities. Certain cross-sectional studies also demonstrated an association between clinical and tooth loss aspects and the presence of visible biofilm [19], dental cavities, and periodontitis [23]. Actually is important invest in a health promotion strategies to prevent oral health disease and the consequence, that is tooth loss, because of the impairment involved with this condition. This is due to the fact that verification between factors was only associated with variable clinics without an association with socio-economic factors. One of the factors that could explain this finding would be the homogeneity of the sample and its socio-economic level, which were also observed in homogeneous samples in adult workers [19] and indigenous people [23]. In our study, the sample displayed a good socio-economic condition. In addition, the municipality has a high Municipal Human Development Index of 0.785. This sample characteristic may also justify the lack of association between tooth loss and socio-economic factors in our study, despite a systematic review with meta-analysis that showed an association between tooth loss and income [40].

In developed countries, such as the United States [9] and in European countries [33], cohort studies have identified socio-economic factors, behavioural factors, and lifestyle to be risk factors for incidence of tooth loss. However, it is not understood if these links are due to a decline in the prevalence of oral diseases or the improvement of socio-behavioural risk factors. It appears that socio-economic inequalities in relation to tooth loss persist, even in developed countries [6], mainly because the reduction in the incidence of tooth loss can highlight socio-economic inequalities [18]. In Brazil, although no longitudinal studies in adults have been conducted to compare results, the composition of the DMFT index, prevalence of missing teeth (M) in the poorest regions of the country and filled teeth (F) in most developed regions in the country were observed [20]. According to Peres et al.[18], these mostly acceptable Brazilian oral health conditions could be explained by improved socio-economic conditions, such as education and improvement in the health system, water fluoridation and use of fluoride in toothpaste. Future longitudinal studies in the adult population should evaluate the context of each component of DMFT that measures cavity experience and especially if public health policies have an impact on reducing oral health inequalities. The absence of oral health cohort studies in developing countries, such as Brazil, reinforces the need to encourage future studies, since these countries may present distribution patterns and risk factors different from what is established in current literature, since they are based on data from developed countries.

Furthermore, in Brazil, the National Oral Health Policy was implemented in 2004, known as “Smiling Brazil,” to meet the entirety of health care within the Unified Health System. Over the last decade, there has been an expansion of public dental service access to the population throughout the country; however, only a minor impact has been reflected in the adult group, as demonstrated in the present study. These results show that some aspects should be reconsidered to meet the needs of this population. Health services need to promote user autonomy and co-responsibility for their own oral health, healthier choices and improved oral health behaviours—all aspects to be established through primary health care. However, health units, which are the appropriate places to perform these types of approaches, operate in hours that are restrictive for the age group of economically active individuals. Therefore, an extension of operating hours or the creation of alternative schedules is required. An additional aspect to be considered is the need for professional practice based on health promotion and prevention of oral diseases, particularly dental caries, so that the decision to extract a tooth is only performed if absolutely necessary [10].

The risk factors for tooth loss were reason for seeking dental services by pain, previous tooth loss and dental caries, that is, the presence of untreated caries associated with late dental service demand determined tooth loss. It should be observed that the risk factors found in our study are modifiable and demonstrate a need for enhancement of health promotion and prevention of oral diseases in public health, especially for dental caries, a disease that can be prevented. In present study socio-economic conditions were not risk factors for tooth loss, however worse socio-economic conditions interferes at risk factors identified in this present study; for example, dental caries and seeking dental service. Thus, strategies aimed at reducing tooth loss need to reduce the experience of oral diseases and their sequelae, especially dental caries. Moreover, different mechanisms need to be addressed in public health policies to combat inequalities in oral health, for example; creating policies that also benefit older age groups and encourage the regular use of oral health services, where health promotion practices are valued as much as assistance to those with this condition.

## Supporting information

S1 File Study Database ‘Prospective cohort of adult oral health in Piracicaba, SP, Brazil’, Brazil, 2015. (XLSX)
