# Correction: Risk factors for tooth loss in adults: A population-based prospective cohort study

**DOI:** 10.1371/journal.pone.0226794

**Published:** 2019-12-16

**Authors:** Manoelito Ferreira Silva Junior, Marília Jesus Batista, Maria da Luz Rosário de Sousa

The following grant number is missing from the Funding statement: Process FAPESP grant 2019/14899-1.
